# Ionic Transport Properties of P_2_O_5_-SiO_2_ Glassy Protonic Composites Doped with Polymer and Inorganic Titanium-based Fillers

**DOI:** 10.3390/ma13133004

**Published:** 2020-07-06

**Authors:** Maciej Siekierski, Maja Mroczkowska-Szerszeń, Rafał Letmanowski, Dariusz Zabost, Michał Piszcz, Lidia Dudek, Michał M. Struzik, Magdalena Winkowska-Struzik, Renata Cicha-Szot, Magdalena Dudek

**Affiliations:** 1 Faculty of Chemistry, Warsaw University of Technology, Noakowskiego 3 Str., 00-640 Warsaw, Poland; alex@ch.pw.edu.pl (M.S.); letmanowski@gmail.com (R.L.); darekzabost@wp.pl (D.Z.); mpiszcz@ch.pw.edu.pl (M.P.); 2 Oil and Gas Institute-National Research Institute, Lubicz 25a Str., 31-503 Cracow, Poland; dudekl@inig.pl (L.D.); cicha@inig.pl (R.C.-S.); 3 Faculty of Physics, Warsaw University of Technology, 75 Koszykowa Str., 00-662 Warsaw, Poland; michal.struzik@pw.edu.pl; 4 Łukasiewicz Research Network-Institute of Electronic Materials Technology, 133 Wólczyńska Str., 01-919 Warsaw, Poland; magdalena.winkowska@itme.edu.pl; 5 Faculty of Energy and Fuels, AGH University of Science and Technology, Av. A. Mickiewicza 30, 30-059 Krakow, Poland; potoczek@uci.agh.edu.pl

**Keywords:** protonic composite conductors, medium temperature PEM fuel cell, sol–gel, phosphosilicate glass, PVA, PEO polymeric additives, TiO_2_, FTIR, FT-Raman, EIS, AC electrical impedance spectroscopy, dispersion of dielectric losses, transport properties

## Abstract

This paper is focused on the determination of the physicochemical properties of a composite inorganic–organic modified membrane. The electrical conductivity of a family of glassy protonic electrolytes defined by the general formula (P_2_O_5_)_x_(SiO_2_)_y_, where x/y is 3/7 are studied by Alternating Current electrochemical impedance spectroscopy (AC EIS) method. The reference glass was doped with polymeric additives—poly(ethylene oxide) (PEO) and poly(vinyl alcohol) (PVA), and additionally with a titanium-oxide-based filler. Special attention was paid to determination of the transport properties of the materials thus modified in relation to the charge transfer phenomena occurring within them. The electrical conductivities of the ‘dry’ material ranged from 10^−4^ to 10^−9^ S/cm, whereas for ‘wet’ samples the values were ~10^−3^ S/cm. The additives also modified the pore space of the samples. The pore distribution and specific surface of the modified glassy systems exhibited variation with changes in electrolyte chemical composition. The mechanical properties of the samples were also examined. The Young’s modulus and Poisson’s ratio were determined by the continuous wave technique (CWT). Based on analysis of the dispersion of the dielectric losses, it was found that the composite samples exhibit mixed-type proton mobility with contributions related to both the bulk of the material and the surface of the pore space.

## 1. Introduction

Various types of fuel cells are attractive energy sources for applications ranging from small-scale portable devices to automobiles and stationary uses. Proton-exchange membrane fuel cells (PEMFCs) are among the types attracting a great deal of interest, as they are capable of producing high power densities while operating at low temperatures. However, the operation of PEMFCs above 100 °C would be an attractive technological solution given the higher tolerance of platinum catalysts for carbon monoxide (CO) and sulphur-containing hydrogen contaminants, faster electrode kinetics, greater energy efficiency, and simplified heat management [1].

Unfortunately, most of the popular polymer electrolyte membranes, such as the Nafion, reveal reduced proton conductivity above 100 °C due to dehydration processes occurring in these materials. Consequently, a snowballing process, including increased ohmic drop occurring in a partially dehydrated membrane, increased generation of Joule heating effects, and subsequent increases in the temperature of the material, leads not only to deterioration of the cell’s operational features but also to premature or even catastrophic wear of the membrane. Maintenance of a suitable level of humidification of all membranes in PEMFC stacks is a very important factor, especially in the case of a stack constructed in ‘open-cathode mode’. In such a configuration, the membranes are air-cooled during operation with dry hydrogen gas stored under pressure in a composite tank [2,3]. In order to improve the performance of power sourcing involving a PEMFC stack with the aim of extending its lifetime or overcoming its slow dynamics during start-up, the humidification process should be also addressed. In the case of PEMFC stack operation, electronic solutions based on a short-circuit unit (SCU) are often applied to control the humidification level in Nafion™-based membranes. Their basic purpose is to produce water inside the fuel cell by means of the electrochemical reaction related to short-circuit current flow. On the other hand, the operating SCU system may also have a negative impact (due to ‘voltage dip’) on other peripheral devices integrated into the hybrid power system [4,5]. To avoid this drawback, various countermeasures were applied to membrane design, enabling reduced sensitivity to humidity level as well as operation at temperatures higher than 100 °C. At this temperature, reaction kinetics are faster, and less expensive catalyst can be added to electrodes. These include the application of novel polymeric backbones such as polybenzimidazoles (PBI) [6], sulphonated poly(ether ketone)s (sPEEK) [7], polysulphones [8], poly(phenylene)s (sPP) [9], or poly(phenyleneoxides)s (sPPO) [10], along with various polymeric mixtures including blends of non-miscible polymers [11,12] and copolymers [13] or polymer-inorganic filler composites. The materials forming the latter group may be based on Nafion™ [14], PBI [15], sPEEK [16,17,18], sPS [19], or sPPO [20]. In case of membrane electrode assembly (MEA) for direct methanol fuel cells (DMFC), Nafion/zirconium phosphate composites according to results of Yang and coworkers, may operate close to 150 °C [21], however, despite these numerous attempts, cell operation well above 150 °C but under 200 °C remains challenging and has not been operationally solved to the best of our knowledge. Therefore, the design of temperature-resistant composite glassy or glassy-ceramic material satisfying these requisite operating conditions in a mid-temperature regime is a worthwhile effort. This has been challenging and important issue for numerous scientific groups for many years [1,22,23,24,25,26,27] Thermal resistance and proton conductivity can be achieved at the same time via several methods: (i) polymer modification by means of grafting of the inorganic acid molecules; (ii) inorganic matrix synthesis with subsequent immobilisation of e.g., phosphoric acid solution; and (iii) (the most promising) synthesis of the inorganic matrix exhibiting intrinsic proteomic conductivity (i.e., phosphosilicate-glass class materials). Therefore, the rationale behind the characterisation (following synthesis) of phosphosilicate electrolyte materials is based on their capacity for serving as solid membranes which, when applied in a fuel cell operating in a mid-temperature regime, maintain the expected proton exchange rate [28,29]. Membranes formed from a phosphosilicate matrix consist of two main constituents, P_2_O_5_ and SiO_2_, forming a homogenous structure of two interpenetrating sub-lattices present in the system [27]. Both originate from the hydrolysis of the appropriate orthosilicate and phosphate organic derivatives, followed by the thermally-driven condensation stage occurring due to water evaporation [25,26]. In the latter process, the monomeric particles are converted into molecules of the corresponding polyacids. These two processes, when combined, lead to the formation of a hydrogel.

The inorganic polymeric network of xerogel is finally formed when the process of dehydration is continued during thermal annealing, which is the last stage of the process used to form the final material. The composition of the final product represents a compromise between mechanical properties (SiO_2_-based sublattice) and protonic conductivity (P_2_O_5_-based sublattice). Moreover, additives such as ceramic fillers affect both the electrical and mechanical properties of the system, due to the activity of their proton-exchange-capable surface groups and their influence on the internal mezzo-structure of glass. A related point is that, in addition to the composition, another phenomenon, porosity, must be regarded as critical to further application of the material and is correlated with pore structure. On one hand, an increase in porosity may improve the value of ionic conductivity, as highly conductive paths are created on the interfacial boundary [26,30,31,32], while on the other, porosity may be a reason for gas leakage in the trough membrane, resulting, finally, in damage to the fuel cell utilising it. Therefore, detailed investigation of two crucial phenomena is essential for improvement of glass membranes. The first is the conductivity or, broadly speaking, the charge transport within the material, which is related not only to the sample’s composition and temperature, but also to its method of preparation, thermal history, and, correlated with these, degree of hydration. Electrochemical impedance spectroscopy (EIS) is a widely recognised tool for the determination of the charge carriers transport properties of various ionically conductive systems. Many research groups have shown its usefulness to study systems like solvent-free polymeric membranes [33,34,35], as well as the hydrated Nafion [36] and sulfonated PEEK [37]. The same method can be, applied to ion exchange properties of the polymeric systems [38,39] and the determination of the bulk and interfacial phenomena occurring in the ceramic ionic conductors [40].

Therefore, basic conductivity studies performed by EIS method should be accompanied by the analysis of conduction activation energy, performed both directly and indirectly, utilising such mathematical formulations as the Meyer–Neldel rule [41,42] and Jonscher’s power law [27,43]. The latter integrates dielectric relaxation data with DC conductivity variations.

The above-mentioned studies should be correlated with investigation of the material’s pore space parameters. These can be analysed in the first approach in terms of the internal specific surface of the glass. Without the application of these intermediaries between the sample composition and its properties, their correlation, and hence their potential for prediction of the deviations of the impedance spectra based solely on the composition, would be impossible. Thus, detailed studies of the influence of the additives on the pore space structure are essential to obtain more detailed information on the limiting phenomena of the process of interest [23,29,44,45,46,47,48,49].

## 2. Materials and Methods 

### 2.1. Synthesis of Materials

**Figure 1:**
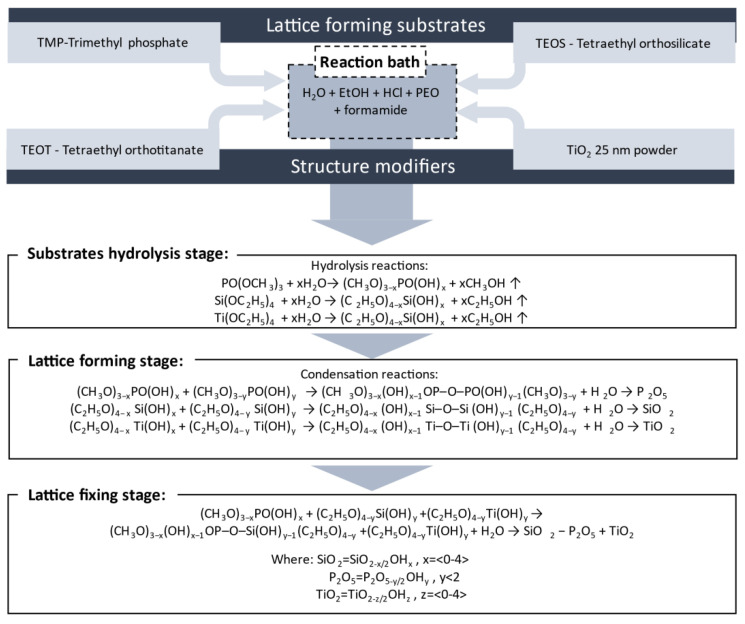
Schematic block diagram of the glass synthesis processes.

The 30P_2_O_5_-70SiO_2_ glass family was synthesised according to the previously described sol-gel procedure with the addition of TiO_2_ and poly(vinyl alcohol) (PVA) (MilliporeSigma, Saint Louis, MI, USA), described in [27]. The samples doped with poly(ethylene oxide) (PEO) were synthesised in a similar way, with addition of PEO (M_w_ = 1 M and 200 k), supplied by Sigma/Aldrich (MilliporeSigma, Saint Louis, MI, USA). In all cases, tetraethyl orthosilicate (TEOS) (Fluka, Buchs, Switzerland, ≥99.0%) and trimethyl phosphate (TMP) (Acros, Geel, Belgium, ≥99.0%) served as the organic precursors. The solvent was a mixture of water (MilliporeSigma, Saint Louis, MI, USA, purified) and ethanol (POCH, Gliwice, Poland, 99.8%) laced with hydrochloric acid (POCH, Gliwice, Poland, analytical grade) and formamide (Fluka, Buchs, Switzerland, 99.5%) The synthesis was conducted according to the synthesis scheme depicted in Figure 1.

The PEO-polymer-doped samples were modified with the addition of Ti atoms containing compounds in a manner similar to modification with the PVA additive [27]. The same atomic fraction of Ti was incorporated into the glass structure, either as a separate phase (TiO_2_ nanopowder 25-nm anatase phase, Degussa, Essen, Germany, P-25 grade) or by means of the direct incorporation of the Ti atoms into the glass structure. In this case Ti(OEt)_4_ (TEOT, Fluka, reagent grade) served as a co-hydrolysed dopant. Due to the faster (compared to TEOS and TMP) kinetics of the TEOT, anon-crystalline intermediate compound (TiO_x_(OH)_4 − 2x_) (ultimately incorporated into the structure of the xerogel) was obtained in situ, but only within the final stage of the main sol-gel process. Water-soluble PEO or PVA macromolecules were selected from a wider range of tested polymeric modifiers. Both compounds are well known for their ability to reduce the internal friction forces of various materials on the molecular level. Their incorporation into the gel in formation was found to be a factor in achieving the greatest improvement in the mechanical properties of the final solid sample. This feature was related to their activity as stress-releasing factors during the stages of sample drying and thermal annealing. On the other hand, being prone to the depolymerisation reactions that occur in acidic conditions, they were easily removed from the sample structure during the pre-measurement annealing stage of sample preparation. Thus, it is worth stressing that, following a period of exposure of the membrane to air or oxygen within the working temperature range, these organic compounds were no longer present.

### 2.2. Analytical Methods of Evaluating Samples

#### 2.2.1. Molecular Spectroscopy

Samples annealed at temperatures ranging from 120 to 200 °C were characterised by Fourier transformed infrared spectroscopy (FTIR) and FT-Raman spectroscopy in order to confirm the chemical stability of the membranes at temperatures close to their working temperatures. A Thermo Scientific Nicolet 6700 FTIR spectrometer with an NXR FT-Raman module (Thermo Fisher Scientific, Madison, WI, USA) with a 1064-nm laser excitation line was used for this purpose. FTIR analyses were conducted in accordance with the attenuated total reflectance (ATR) technique using a Smart Miracle ATR with a Ge measuring crystal, as well as with the diffuse reflectance technique (DR) in mid-IR range (PIKE MIRacle^TM^, EasiDiff, Pike Technologies, Madison, WI, USA). Powdered samples were prepared for ATR measurements; 0.5 wt% of glass in KBr matrix mixtures with no application of pressure was used in the case of DR measurements. FT-Raman analysis was performed using 256 repetitions with a laser power of 0.7 mW, adjusted optimally to avoid thermal effects on the sample.

#### 2.2.2. Porosimetry and Microstructure

The specific surface area was determined based on the Brunauer–Emmett–Teller (BET) equation using a Micromeritics ASAP 2010 nitrogen adsorption apparatus (Micromeritics, Norcross, GA, USA). For chosen samples, pore size distributions (PSD), were calculated with the use of the Barrett–Joyner–Halenda (BJH) theory, basing on isotherms measured on a Micromeritics TriStar II 3020 porometer (Micromeritics, Norcross, GA, USA) at liquid nitrogen temperature.

#### 2.2.3. Mechanical Properties

The mechanical properties of the samples were determined by means of the continuous wave technique (CWT), which relies on mechano-acoustic measurements, using a device supplied by Core Laboratories (CoreLab Instruments, Tulsa, OK, USA). This technique relies on the measurement of ultrasonic standing wave resonances in a composite resonator containing the material undergoing examination, achieved here by means of sweeping the excitation frequency over a range wide enough to cover several standing wave resonances. Consequently, the outgoing signal received by the second transducer is modulated and can be broken down into frequencies characteristic of resonances of interest [50]. Values of Young’s modulus and Poisson’s ratio were determined using this method.

#### 2.2.4. Thermal Analysis

Differential thermal analysis (DTA) and thermogravimetry (TG) were used to determine the thermal properties of the samples studied within a temperature range of 25–900 °C in inert gas or ambient air. A Pyris 1 thermal analyzer (PerkinElmer, Waltham, MA, USA) equipped with platinum pans was used for this purpose.

#### 2.2.5. Electrical and Electrochemical Measurements

Electrical impedance spectroscopy (EIS) measurements were carried out using a set based on a Solartron 1260 Gain-Phase/Impedance Analyzer equipped with a heating system (Solartron Analytical, Hampshire, UK). The temperature range of the experiment was set from room temperature to 200 °C in a heating–cooling experimental cycle. For each temperature of the EIS experiments the equivalent circuit elements value was fitted in order to determine impedance value, using Firdavn analytical software by J. Dygas [51]. The method is successfully used in order to examine the ionic conductivity of membrane electrode assembly (MEA) elements [52,53].

The accuracy of the temperature stabilisation was 0.2 °C. The frequency range of interest was from 100 mHz to 10 MHz. Prior to performance of the impedance measurements, the samples were thermally prepared by means of an additional 3-day process of drying at gradually increasing temperatures up to 120 °C. The impedance spectroscopy measurements were carried out in an air atmosphere without the use of any additional humidification systems. Subsequently, opposing flat surfaces of the samples (with a surface area of about 0.5 cm^2^ and a thickness of 1 mm) were polished with fine SiC abrasive paper up to 2000 grit. Next, platinum contact electrodes were attached to both sides by means of DC plasma sputtering. The edges of the samples were then polished again to avoid electrical contact between the platinum electrodes.

The values of electromotive force (EMF) of the elaborated hydrogen concentration cells were as follows:

1. H_2_(I), Pt/C|30P_2_O_5_-70SiO_2_ + 0.5% PVA |Pt/C H_2_(II);
2. H_2_(I), Pt/C|30P_2_O_5_-70SiO_2_ + 0.5% PVA+ 5% TiO_2_ (TEOT)|Pt/C H_2_(II).

These values were used to determine the ionic transport number. Pure humidified hydrogen and a humidified mixture of 10% H_2_ in Ar (flow ~30 mL/min) were used as feeding streams. The EMF was measured by multimetrs Agilent 34411 (6 ½ digit) (Santa Clara, CA, USA)

#### 2.2.6. Methods of Data Analysis Used for Measurements of Electric Properties

The data originating from EIS response at various temperatures were analysed further by means of the Meyer–Neldel rule and dielectric relaxation analysis. The former enabled a detailed study of the correlation between the activation energy and the order–disorder transition temperature occurring in the investigated material [45,54]. It was found that, for a wide range of polymeric ionic conductors [55,56], the magnitudes of the pre-exponential factor (σ_o_) and the activation energies of conduction (E_a_) (both described by the Arrhenius equation)

(1) $$\sigma \left(T\right)={\;\sigma }_{0}\exp\left(-E_{a}/\mathrm{kT}\right)$$

were related via the linear Equation (2).

(2) $$\ln\sigma_{0}={\;\alpha E}_{a}+\;\beta$$

The pre-exponential factor, on the other hand, can be described by the following equation:

(3) $$\sigma_{0}={K\upsilon }_{o}\exp\left(\frac{{\Delta S}_{m}}{k}\right)$$

where K is the correlation term, k is Boltzmann’s constant, υ_0_ is the ionic oscillation frequency, and ΔS_m_ is the entropy of ion migration. For a range of materials, the value of the entropy of ion migration ΔS_m_ and the enthalpy of activation (E_a_) are related to the order–disorder transition temperature (T_D_) in accordance with the following equation:

(4) $$\frac{E_{a}}{T_{D}}={\;\Delta S}_{m}$$

The high-frequency dielectric response of the electrolyte was analysed via the application of Jonscher’s power law of universal dielectric response [43]. For this purpose a log(ω) − log(σ_re_) representation was used. Jonscher’s law enabled the computation here of the frequency hopping of the charge carrier (ω_p_) on the basis of high-frequency impedance, using Equations (5) and (6).

(5) $$\sigma_{\mathrm{re}}\left(\omega \right)={\;\sigma }_{\mathrm{DC}}+{A\omega }^{n}$$

(6) ωp=(σDCA)1n

where:

σ_re_— real part of the susceptibility (AC conductivity) of the sample;
σ_DC_—direct current conductivity of the sample;
A, n—material parameters;
ω—alternate current frequency.

The value of the material parameter n varies for different materials within the range 0 to 1. When n is within the range 0.5 to 1, the real part of electrical susceptibility is higher than the imaginary part. For the range 0 to 0.5 the reverse is true. For n = 0.5, both parts are equal. A case where n = 1.0 cannot be achieved for real materials (as it represents the case of the ideal dielectric) and in practice can be reached only as a result of an experimental error for low-loss materials.

Therefore, it was found that the representation of relaxation-based properties achieved through the calculation of Jonscher’s corresponding initial and secondary parameters could be correlated with the morphology of the material, in particular with the value of its specific surface area, and thus with its porosity. The correlation proposed here was to bind the value of the n parameter in Jonscher’s equation (which is the measure of the dielectric non-ideality of the system) with the dimensionality (d) of the conductivity process.

(7) d=2+n 

In an idealised case of loss-free bulk-only conductivity, the value of d is 3. For porous solids where conductivity through the internal material surfaces is present, the dimensionality and thus the value of n decreases. With increasing specific surface area of the material, the pore space parameters change, while the surface-related charge transport phenomena become more and more prominent in the overall impedance image of the sample. The theoretical approach applied here may be based on the fractal dimensionality of the geometrical object representing the open internal surface of the porous sample. While the value of the topological dimensionality equals 3 for a solid body, it decreases to lower fractal values if the pore structure is sufficiently complex and developed. Moreover, the impedance response of the nanostructured materials has been widely discussed and approximated by means of various models (a detailed comparison is given in [57]). Independently of the level of model complication, the resulting impedance image reveals the distribution of the time constant for the corresponding resonator. The strength of the deviation here is usually approximated with a non-discrete element characterised by the value of the phase shift, with the exponential parameter n being characterised by the same physical sense as the n of Jonscher’s exponent. For porous materials, this parameter typically falls within the range 0.6–0.95, resulting in dimensionality of conduction equal to 2.6–2.95.

In our previous work [27], it was found that, among the studied samples, those with the lowest porosity revealed values of n equal to approximately 0.9, whereas the most developed micromorphology corresponded to n = 0.7. On the other hand, it was found that the values of the specific surface determined via this method were highly dependent on the preparation of the sample used in the absorption experiments. The greatest deviation was correlated with the modification of the grinding method and size fraction of the resulting grains.

Finally, in addition to the above-mentioned porosity-nonideality correlation, a study of the charge carriers availability was performed using the same mathematical approach. The ‘real’, or, more precisely, ‘effective’ concentration of charge carriers was calculated from Equation (8), describing the spatial density of the sub-population of the actively involved charge- transporting species. This value was typically several orders of magnitude lower than the theoretically available amount of charged species found from the analysis of the sample’s stoichiometry.

(8) $$K=\frac{\sigma_{\mathrm{DC}}T}{\omega_{p}}$$

## 3. Results

### 3.1. Results of Electrical Impedance Spectroscopy

**Figure 2:**
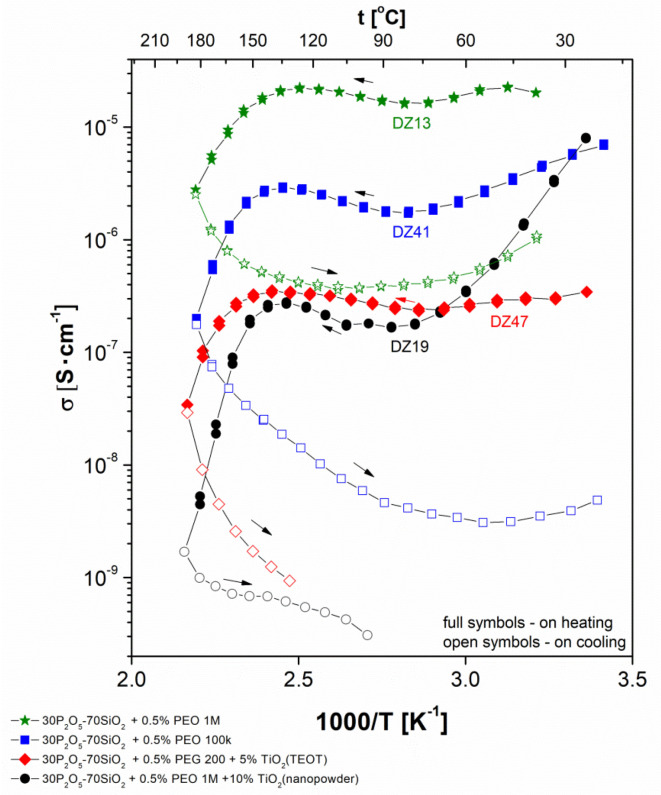
Electrical conductivity dependencies vs. temperature curves recorded in the heating–cooling cycle for the investigated series of poly(ethylene oxide) (PEO)-doped glass compositions: ★—30P_2_O_5_-70SiO_2_ + 0.5% PEO 1 M; ■—30P_2_O_5_-70SiO_2_ + 0.5% PEO 100 k; ♦—30P_2_O_5_-70SiO_2_ + 0.5% PEG 200 + 5% TiO_2_ (TEOT), ●—30P_2_O_5_-70SiO_2_+ 0.5% PEO 1M + 10% TiO_2_ (nanopowder).

**Figure 3:**
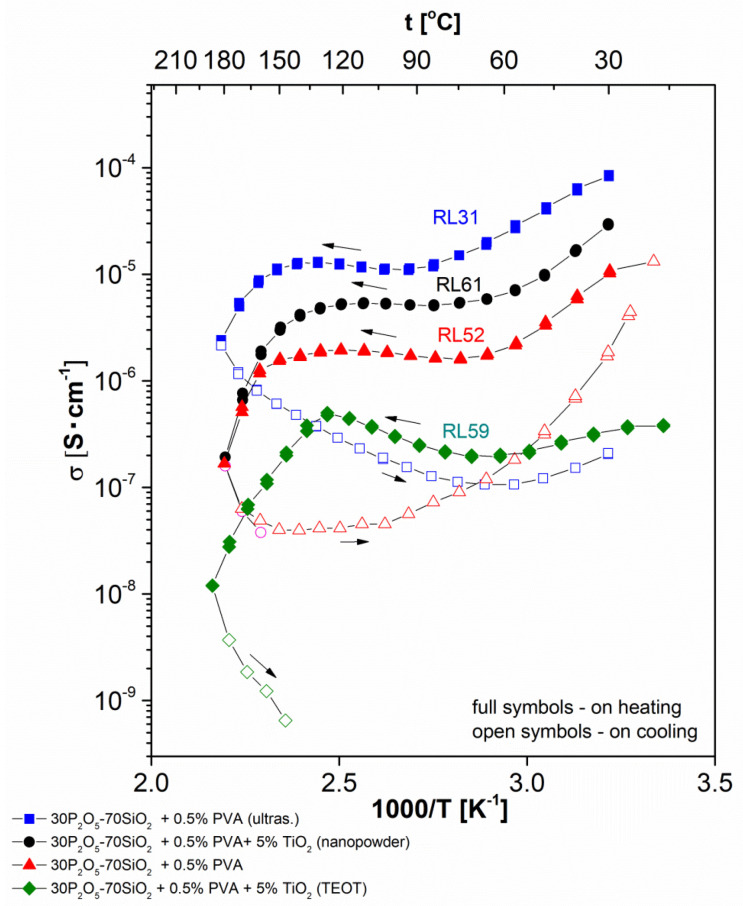
Electrical conductivity dependencies vs. temperature curves recorded in the heating–cooling cycle for the investigated series of poly(vinyl alcohol) (PVA)-doped glass compositions: ■—30P_2_O_5_-70SiO_2_ + 0.5% PVA, (ultrasonificated); ●—30P_2_O_5_-70SiO_2_ + 0.5% PVA + 5% TiO_2_ (nanopowder); ▲—30P_2_O_5_-70SiO_2_ + 0.5% PVA; ♦—30P_2_O_5_-70SiO_2_ + 0.5% PVA+ 5% TiO_2_ (TEOT).

Figure 2 shows thermal variation of total conductivity recorded during the heating–cooling cycle for all studied glass compositions modified with PEO, while Figure 3 shows thermal variation of conductivity for PVA modified system. As the framework of the tested material (two inorganic polymers forming an interpenetrating network) remains rigid within the experimental temperature range, an Arrhenius type of thermal dependence of ionic conductivity would be expected for the examined samples. We observe, however, strong deviation from linear dependence in protonic conductivity as a function of temperature. 

At low (near-ambient) temperature, we observe a significant decrease in conductivity value with heating of the sample. This decrease of conductivity, near to one order of magnitude or less, was observed for all but one sample. In this unique case (glass modified with the addition of PEO and TiO_2_ nanopowder) the value of the conductivity drop was even greater, equalling two orders of magnitude.

This type of behaviour was contrary to the expected one, based on the theoretical predictions. To understand this divergence, strong dependency of conductivity on the material hydration level (or water content in the sample) must be considered. It should be noted as well that the water molecules scavenged in the material structure belong to various subpopulations. They differ in terms of the mechanism, and thus the bonding strength, existing between them and the solid glassy lattice. This, in turn, can be attributed to various ranges of temperature characteristic of the process of their liberation and subsequent expulsion from the material. Within the range of temperatures discussed here (from ambient to 70–80 °C) only weak-bonded (so-called “free” water) was expelled. The molecules addressed here existed either as the liquid phase filling the pore space or were immobilised on the surface of the empty pores via a process of physical adsorption. While the external surface of the specimen increased the available area, in practice it remained at least three orders of magnitude less than the internal surface, and thus in this respect was meaningless. These molecules influenced conductivity significantly, as a result of their high level of mobility and the liquid-like mechanism of the proton transport (see below, in this section). 

In addition to the observed decrease, it must be stressed that the corresponding conductivity values suffered as well from significant instability when a set of measurements was performed repeatedly at a constant temperature. This resulted from the bulk mass transport limitations of the drying process, slowing it to the point where it was unable to reach equilibrium within the period of time associated with the unitary temperature step of the measurement. Moreover, the measured values varied when a set of specimens fabricated from the same type of material underwent succeeding investigations. To understand these observations, it can be assumed that various levels of the initial sample humidity existed for different specimens, even if they derived from the same run of the synthesis process. This value was sensitive to the storage conditions of individual specimens. Fortunately, these instabilities did not affect the properties of the material under working conditions and were limited only to ca. 80 °C.

In continuing the heating sub-cycle, the next range of temperatures was attributed to the expected increase in conductivity upon heating correlated with the more or less stable hydration conditions of the system. The response observed here was linear and fulfilled the assumptions of the Arrhenius theory. This type of predictable behaviour occurred typically within the range 70–150 °C for PVA-doped samples and for this with PEO additive in the range 70–140 °C. The exact values of the border temperatures depended on sample composition; however, dependence was not particularly significant, as the relevant shift did not exceed ±20 °C for either limiting value. A subsequent increase in temperature above its upper limit of linearity in a typical experimental setup where the sample was not additionally humidified in the course of measurements led to further material dehydration. This resulted in a conductivity drop of at least two orders of magnitude. This value covered the range between the last point within the area of the linear Arrhenius-type behaviour and the value of conductivity corresponding to the highest temperature applied (250 °C). This process was only partially similar to the initial drop in that it was related to additional sample dehydration. Contrary to the situation described above, this time the chemically bonded water was partially expelled from the sample’s structure. This range of temperatures corresponded to the range characteristic of the condensation of both orthoacids to the molecules belonging to the families of polyphosphoric and polysilicate acids, which vary in their condensation level.

The shape of the registered dependency was completely different when the measurements were performed during the cooling sub-cycle following the previous heating sub-cycle up to 250 °C. In this case, the shape of the observed curve could be attributed to an incomplete ‘reversed image’ of the previously observed processes. The fragment of the curve missing here corresponded to the phenomenon of high-temperature dehydration. There should have been an increase in conductivity at the initial cooling stage originating from water uptake and bonding by the poly-acids molecules; this was impossible in practice in the case of the applied measurement setup. Therefore, instead of an increase, a monotonic Arrhenius-type decrease in conductivity was observed, reaching a slightly lower temperature in comparison with the threshold of the conductivity increase observed upon heating. Moreover, the conductivity values on cooling were significantly lower than those on heating. The gap between the curve fragments was in a range from 1 to 3 orders of magnitude. This conductivity depletion was irreversible in nature under experimental conditions. In the succeeding heating cycles, the initial high values could not be achieved again. Even more interestingly, this observation remained valid even if the pre-measured samples were later stored in conditions identical to those applied just after annealing (ambient temperature and humidity) for a similarly long period of time, as had occurred previously with the ‘fresh’ specimens. Therefore, the behaviour of the ‘measured’ samples (heated to 250 °C), even if the possibility of regaining material humidity was ensured, differed from the characteristics of ‘fresh’ material, in which water re-uptake processes were present. This observation will be addressed further in terms of other discrepancies observed between these two sample conditions and related to their thermal history.

The activation energies calculated for the two above-mentioned fragments of the dependency for the same samples (presented in Table 1) were, as well, significantly different. Higher activation energy was observed for the cooling sub-stage. The meaning of this phenomenon is addressed in detail later in this section and attributed to changes in the level of hydration of the studied material.

Finally, following cooling within the lowest temperature range, a hydration-related increase of conductivity was observed for most of the samples. This process, observed for the given material at the time of measurement, took place within a temperature range narrower than that associated with the first stage of the initial heating sub-cycle. On one hand, the conductivity increase observed here narrowed the gap between the lowest temperature conductivities to some extent; on the other, as mentioned above, only a partial recovery of conductivity was observed.

The values of the ‘dry state’ (cooling sub-cycle) electrical conductivity ranged from 10^−4^ to 10^−9^ S/cm, depending on the temperature and type of material undergoing the examination. The observed variability range was significantly wider compared to the results gathered for ‘wet’ conditions of samples. In the latter case the maximal value of the conductivity was approximately 10^−3^ S/cm, with observed variability of approximately one order of magnitude. This discrepancy is easy to explain given that proton-hopping is a predominant mechanism in solvent-free conditions, whereas in the ‘wet’ state the so-called ‘vehicle mechanism’ dominates proton transport [28,58,59].

**Figure 4:**
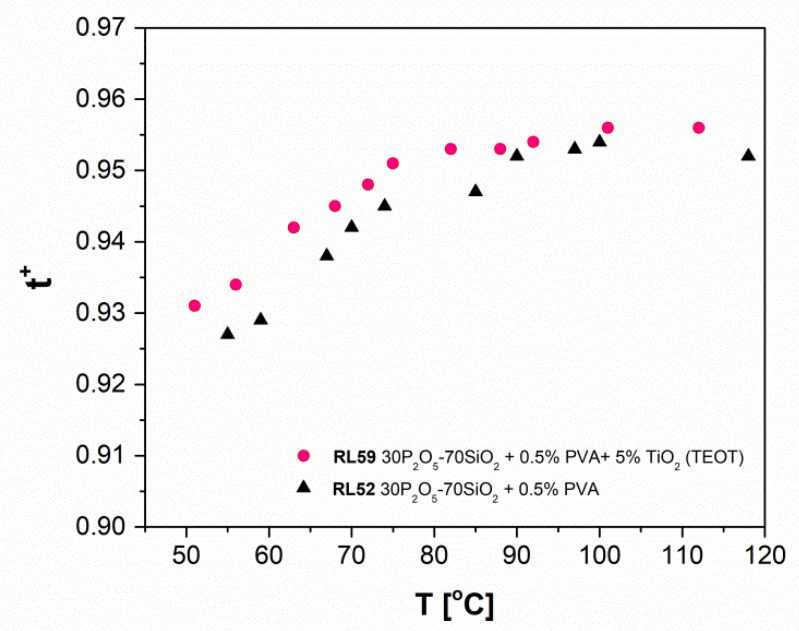
The variation in transference number t_ion_ of samples RL52 and RL59 determined by means of electromotive force (EMF) measurements of a hydrogen concentration cell.

Thus, the charge transport is related to the mobility of the water molecules in the internal structure of the sample and, of course, to their ability to associate and bond protons forming ions with the general composition H_(2n+1)_O_n_^+^. According to various proposed models suitable for systems with various compositions and concentrations of water molecules, the value of n may be equal to 1, 2, 4, or even greater values, as optimal proton mobility in Nafion™-like materials is attributed to the involvement of 10–13 water molecules. The dependency of the mobility coefficient of these species on the n value is correlated to conductivity with the molar ratio of water molecules to mobile protons. As a result, the final value of conductivity is virtually independent of the solid matrix composition. This assumption is valid unless the change in the latter results in no significant differences in the capacity for water uptake and retention, which is dependent in turn on the value of the affinity of water to the matrix of interest, as the two latter parameters determine the equilibrium level of the hydration of the material at a given temperature. Contrastingly, in the ‘dry’ state, Grotthuss-type transport [60,61] long polyphosphoric acid molecules are dominant, and the structure of the glass significantly affects the conductivity value. To build a cause-effect string linking dry conductivity with the type of the material studied, it must be taken into consideration that molecular structure and micromorphology are composition-related properties of the glasses under investigation. Finally, it is worth stressing that variability in the hydration level of the sample may also be the result of differences in storage conditions or, more generally, in the history of the particular specimen of conductive glass. In the initial stage of the experiments we determined a difference of two orders of magnitude in conductivity values between samples characterised by the same composition but deliberately annealed in different regimes, and thus exhibiting various hydration levels. Accordingly, identical treatment of all samples prior to further examination was strictly maintained to eliminate this source of discrepancies. In the Figure 4 the t_ion_ transference numbers were also estimated from EMF measurement of the steam/hydrogen concentration cell:

1. H_2_(I), Pt/C|30P_2_O_5_-70SiO_2_ + 0.5% PVA|Pt/C H_2_(II);
2. H_2_(I), Pt/C|30P_2_O_5_-70SiO_2_ + 0.5% PVA + 5% TiO_2_ (TEOT)|Pt/C H_2_(II).

In these conditions, the transference numbers determined for both samples fell within the range ~0.93 to ~0.95 for the temperature 50–120 °C. 

These data confirmed that the examined samples exhibited dominant protonic ionic conductivity. The results from electrochemical measurements of hydrogen concentration cells stay in agreement with electrical conductivity investigations performed by EIS electrochemical impedance spectroscopy method.

### 3.2. FTIR and FT-Raman Spectroscopy Results 

**Figure 5:**
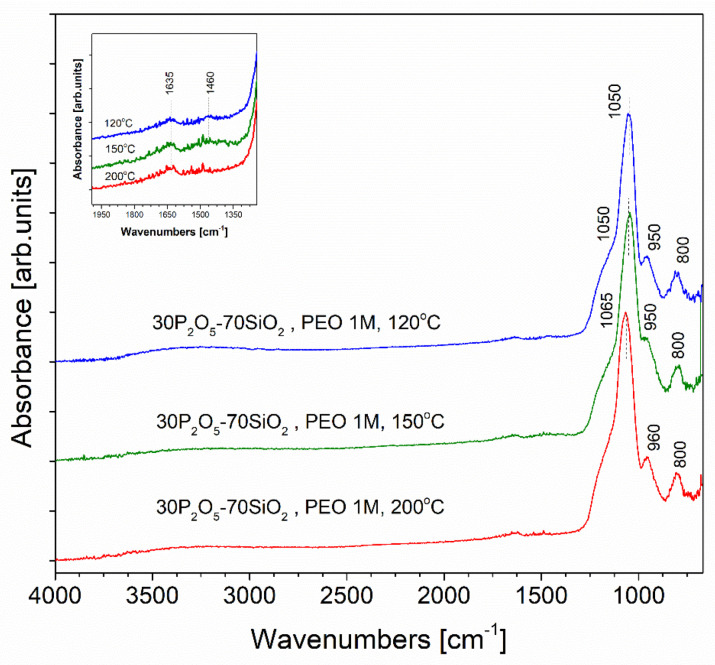
Fourier transformed infrared spectroscopy (FTIR) attenuated total reflectance (ATR) spectra recorded for PEO-doped samples annealed at temperatures of 120, 150, and 200 °C.

In order to study the molecular level structure of the material, the results of infrared and Raman spectroscopies were analysed. In contrast to the significant deterioration of the transport conditions of the charge carriers described above, structural changes related to the heat treatment determined via the analysis of the FTIR spectra were not very pronounced (Figure 5).

It was only for the main asymmetric stretching mode of the Si-O bond that a slight change in the position of the band maximum was observed. Upon dehydration, this position shifted towards higher frequencies, from about 1050 cm^−1^ for the initially dried sample to 1065 cm^−1^ after annealing [62,63]. The observed effect was virtually independent of the type of the organic additive used (according to the data for PEO-modified materials in the present paper, along with those presented previously [27] for PVA-modified materials). Another change in the spectrum related to annealing was observed within the range located in the vicinity of 1460 cm^−1^. A weak and broad absorption band, attributed to the presence of residues of the organic substrates, was present in the initial material spectrum. Its intensity decreased along with increases in the annealing temperature. Finally, this band disappeared when samples were annealed at 200 °C.

**Figure 6:**
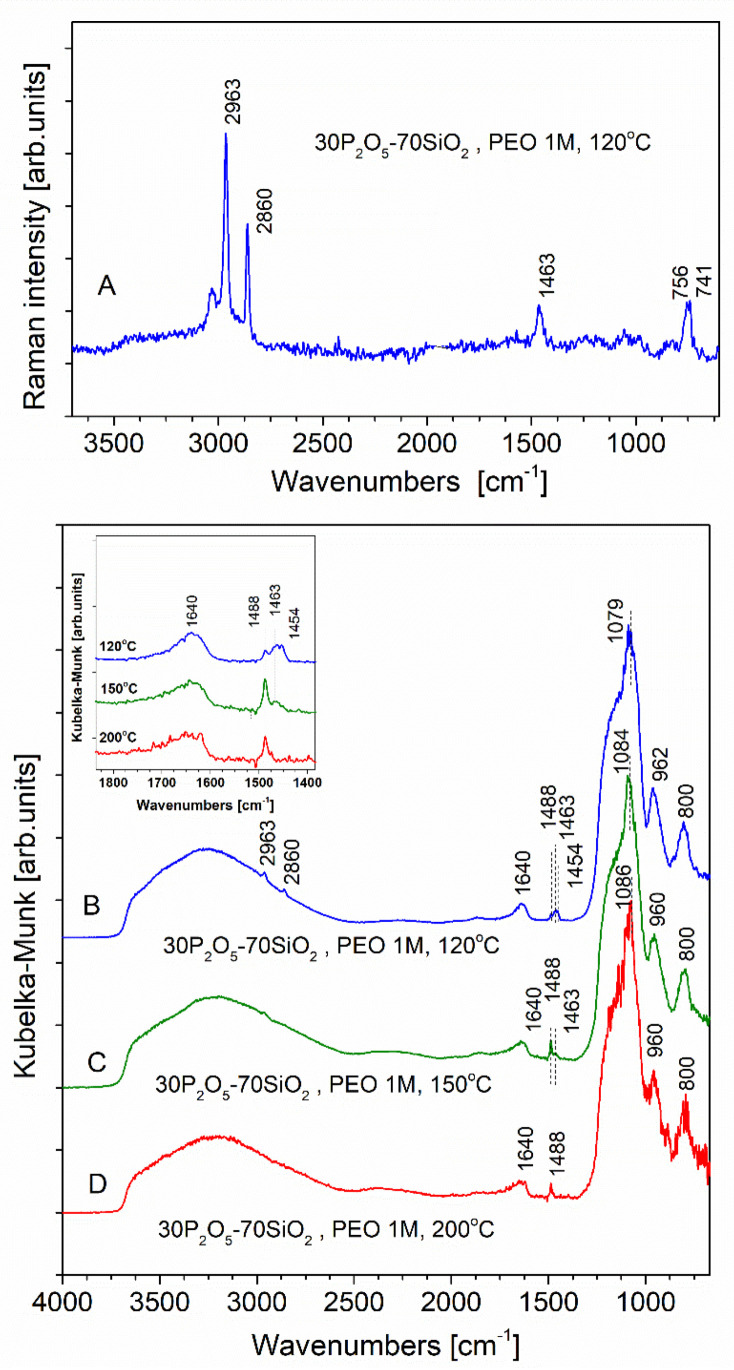
Raman scattering (**A**) and diffuse reflectance FTIR (**B**–**D**) spectra of 30P_2_O_5_-70SiO_2_ glass modified by the addition of PEO M_w_ = 10^6^ g/mol. Spectra were recorded following sample annealing at 120 (**A**,**B**), 150 (**C**), and 200 °C (**D**).

In order to gather additional information, a complementary spectroscopic examination was performed by means of Raman spectroscopy (Figure 6A) as well as FTIR via the diffuse reflectance technique (Figure 6B–D). In the spectra of the studied materials collected with the use of the latter technique, two main differences can be observed in comparison with the previously presented ATR results. Before describing these in detail, it must be stated here that high-quality spectra are not always possible using the DR method. However, the advantage of this technique is good signal-to-noise ratio, especially in the mid- and high-frequency ranges. Thanks to this, it was possible to distinguish two peak maxima: one at 1488 cm^−1^ and the other (broad and even lower in intensity) located in the vicinity of 1463 cm^−1^, with an accompanying maximum in the vicinity of 1454 cm^−1^. These weak but clearly visible bands may be attributed to the presence in the finally formed ‘dry’ glassy material of residual amounts of organic substrates which were not subject to reaction during sol-gel synthesis. 

The other discrepancy is related to absorption bands located in the range 3000–3600 cm^–1^ and in the vicinity of 1640 cm^−1^, which were attributed to both vibrational modes of the hydroxyl moiety. The DR collection technique enabled us to observe both of these bands without the artefact of non-linear response typical of ATR equipment, the presence of which consequently leads to significant weakening of band intensities in the high-frequency range. In addition, due to good intensity response in the region above 3000 cm^−1^, we were able to observe here (in the IR spectra of samples annealed at 120 and 150 °C) (Figure 6B,C) two bands (C–H stretching) originating from trimethyl phosphate molecules with maxima at 2852 and 2963 cm^−1^. In the case of ATR they remained indistinguishable from the broad signal associated with the –OH bands. The latter assignment could be confirmed by the Raman scattering spectrum, which enabled full identification of the non-hydrolysed trimethyl phosphate molecules presented in the same (Figure 6A). This observation is in agreement with data published for similar materials by M. Nogami et al. [46], who reported the presence of unhydrolysed TMP which remained unchanged even in dried gel.

These two bands were present independently of our careful optimisation of the synthetic route. In earlier stages of the synthetic efforts, the major aim of the above-mentioned approach was to level out the kinetics of the two (TMP, slower; TEOS, faster) hydrolysis processes. Where is this goal was mostly achieved, since the intensity of the TMP originating signal was significantly reduced upon optimisation, complete conversion of the less reactive precursor was found to be unachievable. On the other hand, from a practical point of view, the formation of the polysilicate (faster) and polyphosphate (slower) sub-lattices [46] was characterised by virtually the same rate of progress of the reaction.

**Figure 7:**
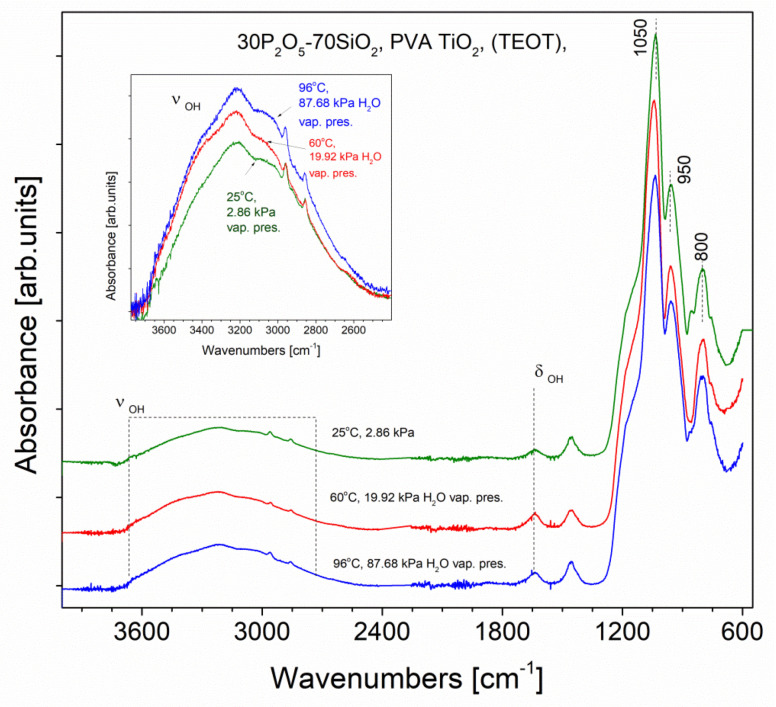
FTIR ATR spectra recorded for the sample RL59 held in an increasing water vapour pressures: 2.86 kPa achieved in a temperature 25 °C with no additional humidification; 19.91 kPa achieved in a temperature of 60 °C and 87.68 kPa in temperature of 96 °C.

The PVA-doped samples previously tested as a MEA (membrane electrode assembly), in the presence of humified hydrogen, have also been examined structurally in similar humidity conditions. As an example, the results for RL59 sample doped with PVA polymer as well as TiO_2_ (TEOT) are presented in Figure 7. The sample was analysed using FTIR ATR method. Prior to molecular spectroscopy examination, the sample was grinded and treated by water vapour of a pressure of 19.92 kPa achieved in 60 °C and after in 87.68 kPa water pressure in 94 °C. On each stage the spectrum was taken. These results were compared with the spectrum of the sample held in a 25 °C in thermodynamic equilibrium with the environment (the water vapour pressure value was 2.86 kPa). In Figure 7, one can see nearly no structural changes of the material except increasing intensity of the OH bands of molecular water proportionally to increase of a water vapour pressure which probably is trapped in the sample pore space.

All the spectra in Figure 7 are normalised to the most intense band in a range 600–4000 cm^−1^, which is Si–O asymmetric stretching vibration. They are also shifted on the graph to show possible differences clearly. We assume this maximum (near 1050 cm^−1^) does not change during the experiment, so using it, we may judge the relative intensity of the bands assigned to the OH groups present in the samples. The range of stretching vibrations of OH groups was enlarged to show the increase of its absorption value during the humidification process. For the purpose, the spectra have also been corrected relative to the baseline.

The most important tentative band assignments for FTIR and Raman spectra have been gathered in Table 2.

### 3.3. Meyer–Neldel Rule Calculations vs. DTA Calorimetric Results

Given the Arrhenius type of behaviour mentioned previously, it was also possible to determine the value of the system’s dienes temperature by applying the Meyer–Neldel rule, used here to perform calculations with the conductivity data originating from both sets of samples, which differed in terms of the type of polymeric additive. For the glassy materials obtained from the sol-gel composition containing PVA, the temperature value fell within the range 430 ± 20 °C for the heating sub cycle. In the case of materials primarily incorporating PEO as an additive, this value was slightly higher: 490 ± 30 °C. Independently of the observed discrepancy, it is noteworthy that these values lay between temperatures of the melting point for two polymorphs of phosphorus pentoxide. These melting temperatures, 340 and 542 °C, are reported, respectively, for two standard so-called ‘O’ polymorphs of this compound, both of which are capable of existence in the studied conditions. In addition, the determined values are consistent with numerous values of order–disorder temperatures characteristic of ionically conductive phosphate-based systems differing in chemical composition as well as in preparation routines [66]. Pristine P_2_O_5_ (in its basic polymorphic form) can be considered here as a reference system for the values of phase transition temperatures occurring in the polyphosphoric-acid-based sub-lattice of the studied glasses. Comparison of its relevant properties with those attributed to it proves that, in multi-component materials, independently of the type of the polymeric additives used as forming agents, an increase in temperature values corresponding to the transitions of the system can be observed. This phenomenon can be explained by the additional immobilisation and consequent stiffening of the polyphosphoric acid chains. One possible explanation attributes this phenomenon to the interactions of two interpenetrating oxide networks constituting the building blocks of the studied materials. Another possible explanation involves partial formation of the ‘O’ polymorph. The latter assumption is significantly reinforced by the potential occurrence of the template effect. Therefore, the templating process of the polyphosphoric chains can be induced by the silicate-based part of the glass. This template function of SiO_2_ originates from the spatial coherence of the silicate-based structures with the ‘O’ polymorph of the P_2_O_5_. Therefore, considering the nature of the transition of pure polyphosphoric acids occurring within the corresponding temperature range, as described in the literature, it can ultimately be assumed that the transition observed in the studied materials leads to a similar rearrangement of the phosphorous sub-lattice into a structural analogue of the silicate framework.

**Figure 8:**
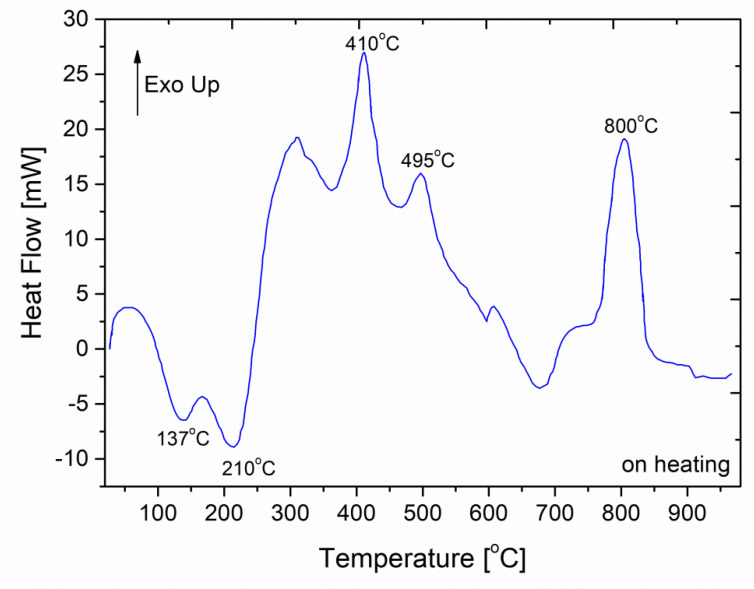
Representative differential thermal analysis (DTA) curve for a glassy electrolyte sample recorded in an ambient air atmosphere.

It was additionally found that the determined values corresponded to the temperature range within which a thermal effect was observed in DTA experiments (see Figure 8) for most of the sample compositions. This phenomenon had not been previously ascribed to any of the phase transitions occurring in glasses of this type. Therefore, taking into consideration the physical meaning of the temperature of the dienes, it was possible to ascribe the observed phenomenon on one hand to the polymorphic transition of the phosphate sub-lattice and on the other to the liberation of the movements of the proton carrying moieties from the limitations related to its spatial arrangement. However, this observation entailed no practical consequences. It must be borne in mind that this would have been possible only if, within the desired temperature range (400–500 °C), the charge transport had been governed by a set of phenomena present in the 70–150 °C range. The assumption made here does not apply, obviously, when the nature of changes (dehydration and further degradation) occurring during subsequent sample heating is taken into consideration.

Thus, determination of the temperature of the dienes based on the data describing the ‘dry’ state conductivity of the studied materials was of value. To this end, the Arrhenius dependencies for the cooling sub-cycle measurements were processed in the same manner, resulting in values approximately twice those mentioned above. For the PEO and PVA pre-modified sets of materials they were equal to 870 ± 30 and 860 ± 30 °C, respectively. Therefore, considering the determined error bars, the T_D_ value in this case should be understood as an independent modifier. This can be readily understood given that, in samples heated to above 200 °C, neither type of polymer additive was still present, due to in situ thermal degradation. Moreover, the temperature values correlated with the order–disorder transition determined for the heating and cooling sub-cycles of the impedance characterisation revealed a difference great enough to additionally confirm the previous assumption that the studied materials, at temperatures above 200 °C, underwent a transition affecting the charge transport process properties in a quantitative manner. In terms of the T_D,_ samples of both compositions behaved similarly, as dehydration apparently reduced the differences that had been present previously.

Similar to the situation described above, an attempt was made to correlate the determined value of T_D_ with a DTA-derived image of the studied materials. Among the observed signals, one located at approximately 800 °C or above was readily found (see Figure 8). The exact determined values depended on the sample composition and the applied scan rate. Thus, the order–disorder transition predicted by this model for the charge-transporting sub-structure of the ‘dry’ glassy material was confirmed by thermal studies of the same material. It should be noted, however, that the calculated value exceeded the range of temperatures within which the raw conductivity data were collected. Thus, the proposed correlation was valuable to a limited extent, as the absence of any additional significant changes in the charge transport conditions had to be assumed in order to legitimise this kind of extrapolation. This condition is equivalent to the statement that, if the material reaches the ‘dry’ state (understood as the state following an irreversible partial loss of conductivity and increase in correlated E_a_ values), continued heating will affect the charge transport such that the characteristic parameters undergo changes while the mechanism itself remains the same.

Initially [27], the strong exothermic effect observed near 800 °C was interpreted as a process of material devitrification. This assumption was supported by the disintegration of the glass specimen occurring in parallel within the same temperature range (observed as well in the DTA pan, as well as in an independent experiment) into powdered material. Finally, during the XRD examination performed on the resulting powders, the above-mentioned process did not occur. There was no trace of the occurrence of ordering/crystallisation in the material structure as the consequence of thermal treatment ranging up to 1000 °C. No difference was noted in the diffraction patterns of the initial glass (in the forms of both solid pieces and powdered samples) or in the yield of the above-mentioned morphological change.

### 3.4. Analysis of Porosimetric Parameters

The data gathered from samples obtained with the use of the PEO modifier are analysed here in a manner similar to that described in our previous paper [27]. Unfortunately, in this case a similar in-depth correlation of porosity-related parameters with dielectric relaxation data was impossible due to the behaviour of these samples during the nitrogen isotherm adsorption analysis, which differed from that of the PVA-containing series. 

The first item of note here is that it was impossible to complete measurements on a monolithic specimen of the material. In the original state of the sample, the nitrogen gas used as a testing medium was incapable of penetrating the bulk of the specimen; therefore, no properly shaped isotherm could be recorded. Thus, the additional step of sample grinding was taken prior to the measurement itself. This operation opened the sample’s internal structures, enabling the nitrogen to reach a previously inaccessible fraction of the pore space. In this way, determination of artefact-free PSD was achieved for all of the examined materials, along with successful estimation of the surface area of these samples.

The atypical behaviour of the studied glasses containing the PEO modifier resulted from the layered structure of these materials. It can be assumed that a closed pore system is predominant near the surface of the sample, whereas the internal parts are filled with an open micro to mesopore system. The micropores, less than 2 nm in diameter according to the IUPAC pore classification [75], are typically characterised by specific surface area values above 100 m^2^/g. The dependence of the pore structure on the distance from the surface of the sample is a result of the faster kinetics of gel condensation and drying in the surface layer. This indicates the importance of close monitoring of the process parameters at all steps of membrane preparation. In conclusion, it can be said that, in fact, access to the latter fraction was opened only during the grinding process.

The observed profile of the arrangement of impermeable/permeable layers may exhibit an additional practical feature for the design of the MEA of the final fuel cell. In evaluating the profile, it should be taken into account that the closed surface-allocated pores cannot be reached by the inert probing N_2_ molecules; moreover, similarly, this kind of sample micromorphology on one hand limits the permeability of molecular hydrogen while the material is working within the fuel cell regime, thus making the MEA gastight; on the other, it does not affect the charge transport process, as the same volume remains available for the charge carriers and their vehicles.

**Figure 9:**
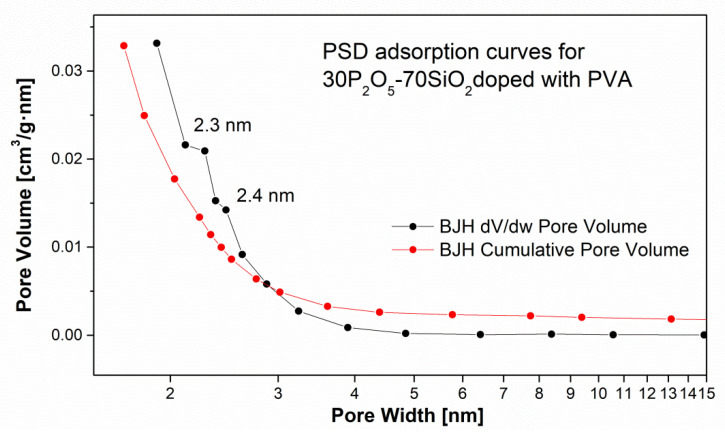
Pore size distribution vs. pore volume (incremental and cumulative) for 30P_2_O_5_-70SiO_2_ + 0.5% PVA + 5% TiO_2_ (TEOT) samples.

**Figure 10:**
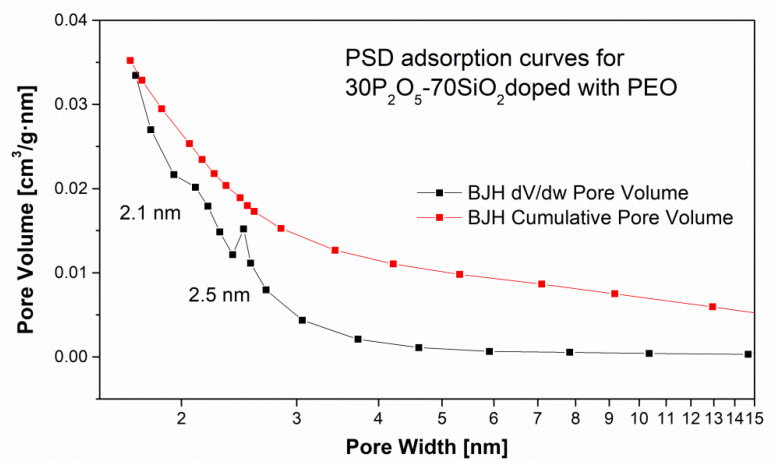
Pore size distribution vs. pore volume (incremental and cumulative) for 30P_2_O_5_-70SiO_2_ + 0.5% PEO 1M samples.

Comparison of the curves depicting PSD for PEO- and PVA-containing samples revealed the presence of meso- and microporous sub-structures in both cases (Figure 9 and Figure 10). Maxima could be observed for pores within a range of 2–50 nm. These results were congruent with analyses by other researchers in comparable systems which reported the presence of pores smaller than 10 nm (especially for the materials synthesised via the sol-gel method) [49,76]. It should be noticed that the surface area determined by the BET model differ significantly between the analysed samples according to the chemical composition. The BET value is 3.7 m^2^/g (for sample doped with PVA and TiO_2_ introduced by TEOT alkoxide)—sample RL59, up to 537 m^2^/g for the electrolyte modified only with 0.5% PEO 1M polymer (sample DZ13).

So, it has been shown that in case of chosen synthetic path densification of the membrane is possible simply by introducing titanium alkoxide and PVA additives into the phophosilicate glass structure.

### 3.5. Comparative Studies: Porosity vs. Dielectric Properties

As described earlier in this paper, the dielectric relaxation-based properties represented by the corresponding initial and secondary Jonscher’s parameters could be correlated with the micro-morphology of the material, in particular with the value of the specific surface area, and thus the characterisation of its open porosity. Therefore, in the next step, an analysis of the high- frequency part of the AC Y_re_(ω) response of the sample was performed with the use of the single parameter relaxation non-ideality model based on Jonscher’s universal power law. The dataset separation used here was largely identical to that applied in the Meyer–Neldel calculations described above, and thus covered the mid-temperature range, where an Arrhenius-like type of sample behaviour was observed. We presented a similar analysis of the results obtained for the PVA modifier and the heating sub-cycle in a previously published paper [27].

Bearing in mind the limitations of the pore structure investigation of the PEO-laced samples, described above, some quantitative observations can be made, confirming the similarity of the behaviour of the tested family of materials to that of those described previously. The sample with the greatest surface area (DZ 13), for which this parameter is equal to 537 m^2^/g, reveals the lowest set of values of Jonscher’s n parameter. In this case, a decrease from approximately 0.9 to 0.75 was observed upon the temperature increase covering the area of the linear Arrhenius response of the sample. On the other hand, the material revealing the lowest surface area values (DZ 19, 252 m^2^/g) was characterised by the highest n values, with an average value slightly above 0.9. Their thermal dependence is much less prominent, as well as of a reverse character. In this case an increase in temperature leads, as opposed to the previous case, to an increase in the n value. 

It should be noted as well that DZ 13 exhibits both much higher conductivity value and significantly lower E_a_ than DZ 19. In comparing these two observations with the discrepancy noted above in the changes in n factor with temperature, it can be assumed that both the higher conductivity value and its less prominent changes upon heating are correlated with a higher degree of dispersion of material dielectric parameters, and therefore with a higher level of disorder present in its internal structure. Moreover, the nature of the more significant changes in the n value observed for the DZ 13 sample suggest that they are probably not related to water loss upon heating, as this would lead to a reduction in the level of the dielectric non-ideality; rather, they can be assumed to result from changes in the internal pore-space-related structure of the materials.

**Figure 11:**
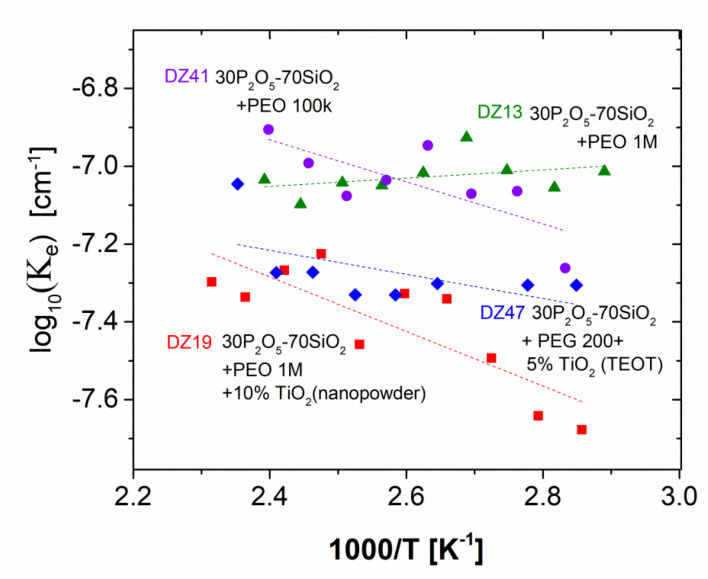
Jonscher’s Ke vs. temperature dependencies with reference to dielectric relaxation data compiled for the investigated series, recorded during the heating sub-cycle of PEO-doped glass compositions: ▲DZ13–30P_2_O_5_-70SiO_2_ + 0.5% PEO 1 M; ●DZ41–30P_2_O_5_-70SiO_2_ + 0.5% PEO 100 k ♦DZ47–30P_2_O_5_-70SiO_2_ +0.5% PEG 200 + 5% TiO (TEOT); ■DZ19–30P_2_O_5_-70SiO_2_+ 0.5% PEO 1 M + 10% TiO_2_ (nanopowder).

A more detailed analysis of the data, performed following calculation of the set of secondary Jonscher’s parameters, led to even more interesting conclusions. During the examination of the effective charge carrier concentration (see Figure 11), it was possible to observe mean values representing a sample increase in the same order as the ionic conductivity of the sample. In the plot, two pairs of samples, characterised by overlapping ranges of concentrations, could be distinguished. The first pair, with significantly higher values, consisted of materials which did not contain the titanium additive. In the other pair, the decrease in value should be probably attributed to the presence of the basic centres localised in titanium. The same order of mean values was observed for ω_p_, i.e., the measure of the charge carrier mobility. Thus, we can conclude with the assumption that the increase in conductivity between materials of various compositions was related to both charge carrier mobility and concentration factors.

**Figure 12:**
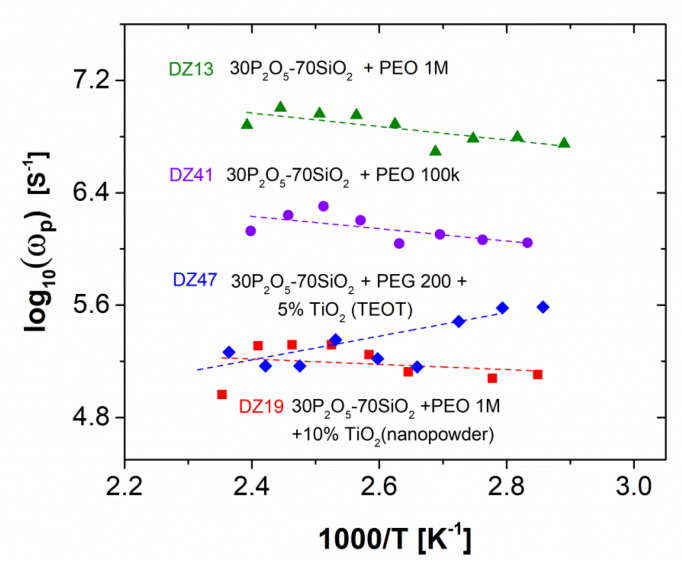
Jonscher’s ω_p_ vs. temperature dependencies with reference to dielectric relaxation data compiled for the investigated series, recorded during the heating sub-cycle of PEO-doped glass compositions: ▲–30P_2_O_5_-70SiO_2_ + 0.5% PEO 1 M; ●–30P_2_O_5_-70SiO_2_ + 0.5% PEO 100 k ♦–30P_2_O_5_-70SiO_2_ + 0.5% PEG 200 + 5% TiO_2_ (TEOT); ■–30P_2_O_5_-70SiO_2_ + 0,5% PEO 1 M + 10% TiO_2_ (nanopowder).

On the basis of thermal changes in the ω_p_ values depicted in Figure 12, it should have been possible to split the overall activation energy into factors, depending on the migrational and creational terms according to the equation E_a_ = E_m_ + E_c_. Unfortunately, considering the low values of the activation energies for the samples, this was virtually impossible due to the uncertainty of the estimation. This could be readily observed in the case of the DZ 47 sample, where a significant increase in the effective concentration of charge carriers upon heating was correlated with a reduction in their mobility factor. The resulting negative value of E_m_ observed for this specimen, as well as the negative value of E_c_ in the case of the DZ 13 material, proved that the charge-transport-related properties of the studied materials were governed by a more complicated set of phenomena compared to those behind the activation-based model of conductivity changes, and thus as well behind the mathematical formalism applied here. Thus, indirectly, the previously assumed significance of changes in the hydration level upon sample heating as an additional parameter affecting the values of the ionic conductivity was confirmed here.

From the deviation from the tendencies expected based on the theory, it can be concluded that this factor influences the conditions of protonic transport to a significant extent, even within the temperature range described initially in this paper as exhibiting ‘stable’ conditions in terms of the water-loss phenomenon. Therefore the ‘stability’ exhibited within this temperature range should be understood as representing a situation in which a sample undergoes changes in hydration level, but the intensity of these changes is limited to a range within which, firstly, no drying-related qualitative changes occur in conduction-limiting processes and, secondly, their influence on conductivity does not dominate but only modifies the observed image.

**Figure 13:**
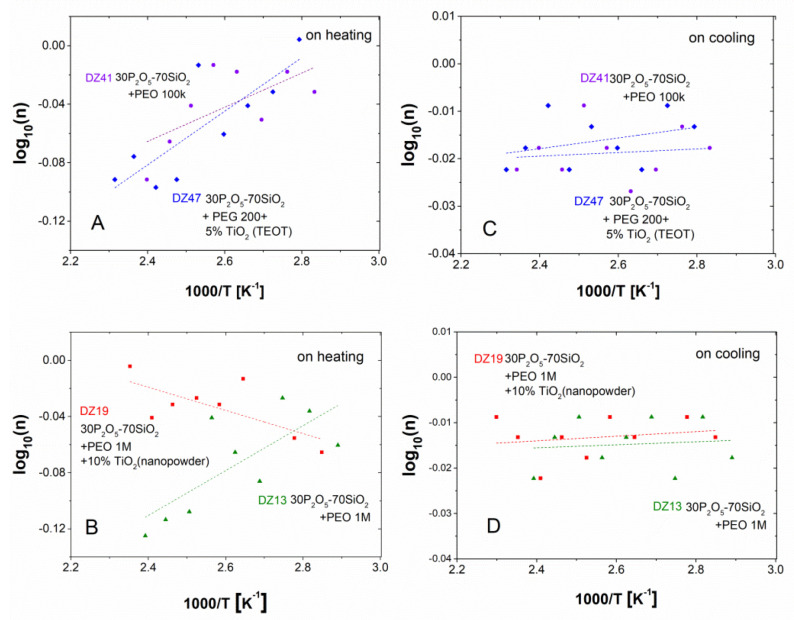
Jonscher’s log(n) vs. temperature dependencies with reference to dielectric relaxation data compiled for the investigated series, recorded during the heating sub-cycle of PEO-doped glass compositions: ▲–DZ 13; ●–DZ 41 ■–DZ 19 ♦–DZ 47.

Subsequent analysis was expanded to include data originating from the cooling sub-cycle. In this case, the same set of samples behaved totally differently, yielding an almost constant value of the n parameter within the range 0.95–0.99, independent of the measurement temperature and the composition of the studied material. The above-mentioned dependencies are gathered in Figure 13. One can easily observe that while the above-said trends are observable, the quality of the linear fit is rather weak. On the other hand, in the studied temperature range, an abrupt change in the n value cannot be observed. Therefore, the material is stable and does not undergo any quantitative transitions. The results are presented on a logarithmic scale. In Figure 13A,B graphs are on heating and C, D graphs relate to cooling cycles. 

This value shows that bulk conductivity was predominant and that non-ideal dielectric behaviour was rather weakly indicated in the results. This behaviour was observed for materials obtained with the use of both types of polymer modifier. This, at first glance, seemed to suggest that this set of observations had yielded no important information. On the other hand, a discrepancy in the dielectric behaviour observed between the heating and cooling sub-cycles may have been correlated with the nature of changes in charge transport conditions occurring upon extended heating. Given that the level of hydration was significantly lower here, the observed increase in the n values to close to 1 corresponded closely to improvement in the dielectric nature or to the reduction in the loss factor of the materials. This obviously occurred parallel to the reduction in the value of conductivity. In addition, the decoupling of the n values from the initial surface area values observed here confirmed the assumption presented earlier in this paper relating the irreversible loss of conductivity observed between sub-cycles to the partial collapse of the pore space present in the material following the structural changes related to dehydration. It must be stressed that the parts of all datasets collected in the cooling mode characterised by the highest frequency revealed the presence of an additional relaxation phenomenon. This kind of dependence extension enables the future application of more developed models of dielectric response, such as the concept of mismatch and disorder introduced by Funke et al. in [77,78]. To perform this kind of calculation, future measurements must be expanded into much higher frequencies.

### 3.6. Results of Mechanical Tests Using the Continuous Wave Technique

Another important trend is indicated by studies of mechanical parameters for selected glassy composites performed using the continuous wave technique (CWT) [50] in the ultrasonic domain (MHz range). The importance of this class of properties cannot be neglected where safety and durability issues of the resulting fuel cell are being considered. The technique applied is well suited (compared to other methods, e.g., pulse transmission techniques) to measurements performed on samples in the form of a thin self-standing film or membrane. The results are compiled in Table 3.

It can be readily seen that both polymeric additives modified the mechanical properties of the resulting glasses in the direction of functional improvement. The intensity of the change was quantitatively represented by the resulting changes in the mechanical properties of the material. In our case, measurements were focused on two parameters: Poisson’s ratio and Young’s modulus.

All of the studied samples underwent investigation following the pre-annealing period. The limiting temperature value at which gradual heating finished was, in this case, 120 °C. As a reference source for the values of material parameters used later for the comparisons, a sample of the pristine additive-free glass of standard composition was characterised in the same experimental conditions. In the next step, the glass with the PVA polymer additive incorporated into the structure was characterised as well. The comparison revealed a decrease in the value of Young’s modulus with the addition of the polymer. This observation can be explained in the light of a shift in the brittle fracture limit [79,80]. The situation was reversed in the case of the polyethylene oxide additive. In this case, the polymeric additive led to an increase in the value of the parameter compared to the above-mentioned reference. Both determined values of Poisson’s ratio also exhibited a change when a similar comparison was performed. Independently, all the values determined here fit the ranges of values typical for various glassy materials.

## 4. Conclusions

The studied materials exhibit a mixed type of proton mobility with contributions related to both the bulk of the material and the surface of the pore space. Previously, we proved [27] that the specific surface of the studied material, which is, as well, an integrating measure of the total open porosity found in a unitary sample, is the parameter of the investigated material which, chosen as an intermediary, enables the correlation of substrate stoichiometry with the resulting material transport properties. In this paper, we additionally prove not only that the porosity–conductivity correlation should be investigated in terms of the average size of the pore (as stated in papers by Nogami [32]) but that the pore size distribution of the sample should be analysed in detail. Moreover, these extended porosimetric characteristics of the material may be juxtaposed with variation in the dispersion of the dielectric loss factor of the material depicted in the frequency domain. The set of parameters describing the phenomenon is relatively easy to determine experimentally when the appropriate model of dependence is chosen to fit the immittance image of the high-frequency response of the material. Thus it was found that without applying a two-step analytical procedure integrating the pore space data into the explanation of the changes in proton mobility it was nearly impossible to achieve a profitable correlation and thus predict deviations in the impedance spectra originating from the non-ideality of the material’s dielectric behaviour. Creation of the reverse correlation was even more difficult due to the insufficiency of data that would have enabled determination of the initial sample composition from the measured impedance responses. It must be mentioned as well that we did not merely examine the electrical properties of various compositions of the conductive glass; changes of conductivity upon drying or hydration of the material also constituted a point of interest. The observations dividing the overall conductivity dependence into three ranges are generally consistent with the assumptions made by Abe and Takahashi [81] suggesting that, along with changes in the degree of hydration of the sample energy barrier for proton hopping, the Arrhenius activation energy of the conduction consequently increases to a significant extent. The proposed deviations are also consistent with the observed range of variability of conductivity. This range was significantly wider in the case of the dry material, from 10^−4^ to 10^−9^ S/cm, whereas the EIS results gathered for ‘wet’ samples cover only a narrow range of conductivity values around 10^−3^ S/cm. On the other hand, our observations are inconsistent with the initial observations made by Tung and Hwang [26]. The existing discrepancies can be explained easily, given the difference in the membrane operational conditions. Finally, it should be noted that the annealing of the composite performed prior to measurement resulted in both mechanical and chemical stability of the electrolyte within the desired range of temperatures corresponding to the assumed working temperature of the hydrogen-oriented MEA, which is the core of the novel mid-temperature design of the fuel cell. The range of changes in the molecular structure was examined here along with conductive stability and, by means of molecular spectroscopy techniques (FTIR and Raman), confirmed as meaningless from the point of view of the requirements of this crucial future application of the studied conducting glasses.

## Figures and Tables

**Table 1:** Activation energies of Arrhenius-type conductivity dependencies for heating and cooling sub-cycles for all compositions of the studied glasses.

Sample Code and Material Composition Description		Ea			
		Heating Subcycle		Cooling Subcycle	
		eV	kJ/mol	eV	kJ/mol
RL 31	30P_2_O_5_-70SiO_2_ + 0.5% PVA (ultrasonificated)	0.092	8.9	0.416	40.1
RL61	30P_2_O_5_-70SiO_2_ + 0.5% PVA + 5% TiO_2_ (nanopowder)	0.059	5.7	0.298	28.8
RL52	30P_2_O_5_-70SiO_2_ + 0.5% PVA	0.093	9.0	0.429	41.4
RL59	30P_2_O_5_-70SiO_2_ + 0.5% PVA + 5% TiO_2_ (TEOT)	0.269	25.9	1.007	97.2
DZ 13	30P_2_O_5_-70SiO_2_ + 0.5% PEO 1 M	0.123	11.9	0.317	30.6
DZ 41	30P_2_O_5_-70SiO_2_ + 0.5% PEO 100 k	0.180	17.4	0.280	27.0
DZ 19	30P_2_O_5_-70SiO_2_ + 0.5% PEO 1 M + 10%TiO_2_ (nanopowder)	0.254	16.9	0.220	21.2
DZ 47	30P_2_O_5_-70SiO_2_ + 0.5% PEG 200 + 5% TiO_2_ (TEOT)	0.116	13.5	0.767	74.0

**Table 2:** Vibration mode assignments for FTIR and Raman spectra.

Vibration Mode Assignments	FTIR ATR Band Maxima Positions (cm^−1^)	FTIR DR Band Maxima Positions (cm^−1^)	FT-Raman Band Maxima Positions (cm^−1^)	References
ν_OH_: (molecular water), Si–OH	3000–3600	3000–3600		[64,65]
νCH_3_ in TMP molecule	2860, 2963	2860, 2963	2860, 2963	[66,67]
δ_OH_: (molecular water)	1635	1640		[64,65]
ν_asym._ (CO_3_)^2−^, (CH_3_O)		1454,1463	1463	[62,67]
ν_asym_ (Si–O–Si), ν_sym_. (Si–O–P), ν_asym_ (PO_4_)	1050, 1160	1085 1150		[25,62,65,68,69] [68],
ν_sym_ Si–O:Si–OH-silanol, ν_asym_(PO_4_)	950, 960	960		[62,64,65,68,69,70,71,72]
ν_sym_ (Si–O–Si), ν(P–O–P)	800	800		[62,64,65,72]
ν(P–O–P), (PO_3_) in TMP, δ_asym_(CO_3_)^2−^	741		756, 741	[73,74] [62]

**Table 3:** Mechanical parameters of representative samples pertaining to various compositions of the studied glasses.

Polymeric Additive Type in 30P_2_O_5_-70SiO_2_ Glass Sample	Young’s Modulus (GPa)	Poisson’s Ratio
no additive	2.73	0.15
additive PVA 85,000–124,000 g/mol in amount of 0.5 (wt.%) of the glass mass	1.67	0.25
additive PEO 1,000,000 g/mol in amount 0.5 (wt.%) of the glass mass	6.09	0.31
